# Supplementary material for: Are carbon emissions trading and green financial instruments synergistic? -Comprehensive quantitative research based on content analysis
Source: PLoS One. 2024 Mar 7;19(3):e0298601. doi: 10.1371/journal.pone.0298601 (PMC10919601; doi:10.1371/journal.pone.0298601)
Supplement: S1 Data — (ZIP) [file pone.0298601.s001.zip › Data/地级市Z_F工作报告.zip!/╣π╬≈/╬α╓▌╩╨/└┤╫╘╣1⁄2╓┌║┼í╛┬φ┐╦╩2╛▌═°í┐_2004.docx]

各位代表：

现在，我代表市人民政府向大会作工作报告，请予审议，并请市政协各位委员和其他同志提出意见。

一、2003年工作回顾

2003年是贯彻落实党的十六大精神，全面建设小康社会的第一年。市人民政府在市委的正确领导下，在市人大常委会的监督和市政协的帮助下，坚持以邓小平理论和“三个代表”重要思想为指导，认真贯彻落实党的十六大和十六届三中全会精神，按照市委提出的“深化改革、扩大开放、提速发展”的要求，求真务实，真抓实干，严格要求，大胆管理，使全市国民经济和社会事业呈现加快发展的势头，全面建设小康社会开局良好。主要表现在：

（一）主要经济指标大幅增长，国民经济呈现加速发展势头。据初步统计，全市完成生产总值163.76亿元，比上年增长10%。其中：第一产业增加值44.29亿元，增长3.5%；第二产业增加值55.35亿元，增长18.4%；第三产业增加值64.12亿元，增长8.1%。全市财政收入14.26亿元（不含基金），增长13.3%。全社会消费品零售总额76.4亿元，增长9.9%；城镇居民人均可支配收入7062元，增长12.4%；农民人均纯收入2007元，增长5.8%。总之，全市国民经济在固定资产投资、工业生产、外贸出口的强劲拉动下，主要经济指标比上年有较大增长，完成情况比预期好，比前几年好。标志着我市国民经济经过几年打基础后，已经进入了一个加速发展的新阶段。

（二）各项改革成效明显，工业生产和效益大幅增长。继续推行国有企业开门改革的成功决策，充分发挥东晖、东旭两家国有资产经营公司的作用，使国有企业改革取得新的进展。去年市政府确定的41家改制企业，已完成产权制度改革或资产重组的国有企业有16家，正在组织实施的企业24家，企业改制面超过全市国有企业资产总量的90%，使非公经济占全市国民经济总量的比重达到50.6%，涌现了以奥奇丽公司为代表的国有企业改革成功的典范。较好地对28家特困企业进行债务重组，共减少债务达3.3亿元，企业生产经营状况得到改善。全市工业投入7.9亿元，其中技改投资完成4.31亿元，增长52.4%，有11个重点技改项目开始试产。全市工业经济呈现出多年来未有的良好局面。规模以上工业总产值达69.5亿元（现价），按可比价计算增长32.7%；完成工业增加值19.75亿元，增长27.6%，规模以上工业企业盈亏相抵后实现利润总额8821万元，增长13.1%，为1998年以来最高水平。工业拉动经济增长3.12个百分点，实现税收比上年增加5608万元。

农业经济结构进一步优化，全市优质稻种植面积占水稻播种面积的比重提高到78.4%，经济作物种植面积占耕地面积的比重达40.5%。水产畜牧业发展较快，瘦肉型猪、三黄鸡等已初步形成产业化发展态势。农村基础设施进一步改善。农村税费改革试点工作取得明显成效，全市农民人均减负49元。财政收入质量明显提高，财政支出结构进一步优化，支持经济发展和城市建设力度加大，政府集中核算支付全面推开。政府职能进一步转变，行政审批制度改革迈出新步伐。

（三）对梧州经济要融入珠江三角洲经济圈逐步形成共识，投资环境明显改善，外向型经济发展良好。通过解放思想再讨论，全市上下对把梧州经济融入珠江三角洲经济圈的重要性、紧迫性有了进一步的认识，更坚定了优势在区位，出路在招商的信心。出台了《关于进一步吸引外来投资促进经济发展的岗位责任制》、《关于实施项目带动经济提速战略进一步扩大对外开放的若干决定》等一批文件，进一步降低了地价、电价、水价，解决了多年来我市招商引资工作中遇到的老大难问题。组织开展联合审批和“一个窗口”收费，建立“主动为外商服务检查汇报制度”、“外商因投资环境问题外迁的部门领导责任追究制度”、“外商投诉督查制度”等，使投资环境明显改善。调整加强了招商专业队，采取强激励严要求的措施调动积极性，较好地促进了全市的招商引资工作。全年实际利用外资8300万美元，增长32.1%，增幅居全区第二位；引进内资实际到位7.24亿元，增长39.2%，创1996年以来最好水平。全市新批三资企业68家，合同投资总额20242万美元，合同外资额11478万美元，分别增长54.5%、264%和210.5%。进一步扩大出口，采取扶持一批外贸企业、扶持一批重点出口产品和扶持一批企业开拓国外市场的“三扶持”措施，下大力气帮助民营企业出口，全市外向型经济高速发展。全年外贸进出口总额2.12亿美元，增长37.7%，其中，外贸出口1.53亿美元，增长31.1%，为1998年以来最高水平。

（四）三百项目大会战势头强劲，固定资产投资大幅度增长。项目建设是改革开放以来形势最好、投资最大、项目最多的时期，群众对开展三百项目大会战给予了很高的评价。大会战已取得阶段性成果，主要表现在：建设项目快速推进，开工的项目达到96项，完成投资19.57亿元，占年度投资总额的96.21%，拉动经济增长2.34个百分点。特别是梧州期盼近20年的长洲水利枢纽工程正式启动，岑溪至梧州高速公路开工，以及河东防洪堤主体工程如期建成并在汛期前发挥作用，极大地振奋了全市广大干部群众的精神，坚定了提速发展的信心。前期工作项目取得重大进展，有91个项目完成年度计划目标任务，其中已开工建设的项目14个，完成前期工作具备开工条件的项目17项。招商项目进展顺利，已签订合同的项目89项，其中有72家办理了营业执照，有63家已投产或开工。

为了把梧州建设成为美丽的山水城市，市委、市政府确定了“一年小变，两年中变，三年大变”的城市建设目标，从根本上改变城市的落后面貌。经过一年的努力，“一年小变”的目标已经实现，较好地解决了市区“灯不亮、路不平、渠不通”的突出问题，城市开始变美了。“两年中变、三年大变”的城市建设项目已经确定，其中骑楼城、西堤路拓宽、新兴三路延长线、鸳江丽港等重点项目已陆续开工。市政建设较好地解决了拆迁户的补偿和安置问题，群众对此比较满意。

在三百项目大会战的强力推动下，我市固定资产投资大幅度增长。全市全社会固定资产投资41.7亿元，增长38.4%，增幅比去年提高23.9个百分点，高于全区平均水平，创我市九年来的最高水平，成为推动我市经济增长的强大动力。其中：基本建设投资、更新改造和其他投资分别增长77%、49.5%和113.8%。

（五）社会各项事业取得新的进步。实施新一轮科技创新计划初见成效。教育事业的投入继续增加，农村中小学校危房改造工作进展顺利，教学条件进一步改善，教育质量不断提高。不断加大对公共卫生设施建设力度，市中心血站、疾病预防控制中心已竣工并投入使用，市传染病医院正在加紧施工，公共卫生和医疗保健工作得到加强。积极做好社会保障工作，“两个确保”继续得到巩固，以养老、失业、医疗保险为主要内容的社会保障体系框架基本确立，城镇居民最低生活保障制度进一步落实。就业和再就业工作取得新进展，全年新增就业16150人，安置城镇失业人员13361人，分别完成计划任务的103.5%和102.8%。全市下岗失业人员再就业交流大会，使3000多名大龄失业人员实现再就业，开创了全区开发公益性岗位安置下岗失业人员工作的先河。金融保险事业健康发展，年末金融机构各项存款余额、贷款余额和各险种投保金额均有较大增长。国土资源管理和环境保护工作力度加大。人口低生育水平保持稳定，人口自然增长率控制在7.77‰以内。体育、文化、新闻出版和广播电视事业健康发展。完成了城郊区和苍梧县的行政区划调整工作。双拥工作取得新成绩，再次荣获“全国双拥模范城”称号。人事、机构、审计、统计、外事、侨务、信访、档案、修志、人防、民族宗教等各项工作又有新进步，老龄、妇女儿童和残疾人等各项事业持续发展。精神文明创建活动深入开展，民主法制建设整体推进。各级政府和部门依法行政水平有新提高。廉政建设进一步加强。国防教育、民兵预备役建设不断加强。全力维护社会稳定，全市刑事案件实现了八年来首次下降，社会治安良好，人民安居乐业。

（六）抗击非典取得重大胜利。面对突如其来的非典疫情，在市委、市政府的正确领导下，在各级、各部门和全市人民的共同努力下，我市抗击非典工作取得重大胜利。在我市每天从广东等疫区回梧人数多达300人、过境人数超过15000人等巨大压力的情况下，通过采取严格的路检、城乡排查、隔离和失职查处等措施，把非典疫情的传播控制到了最小范围，我市除岑溪出现2例输入性非典病例外，其他地方均未发现非典病例，为广西守好了东大门。我市的非典防治工作得到国务院检查组和自治区政府的高度评价。

各位代表，过去的一年，我市国民经济加快发展，社会各项事业取得新的进步，人民生活进一步提高，成绩来之不易。这是全市上下贯彻党的十六大精神，坚持用“三个代表”重要思想统揽全局，指导工作，突出发展这个执政兴国的第一要务，聚精会神搞建设，一心一意谋发展的结果；是我们解放思想，实事求是，与时俱进，实施项目带动经济社会提速发展战略，坚定不移开展三百项目大会战的结果；是我们致力改善投资环境，扩大对外开放，深化国有企业改革的结果；是我们坚持立党为公、执政为民，关心群众生活，一切从群众利益出发的结果；也是广大干部群众转变作风，真抓实干，团结拼搏，辛勤劳动的结果。在此，我代表市人民政府，向全市人民，向驻梧部队官兵，向各民主党派、工商联、人民团体以及海内外所有关心支持我市各项建设事业的各界朋友，致以衷心的感谢和崇高的敬意！

在肯定成绩的同时，我们也清醒地看到，目前我市经济社会发展中仍然存在不少困难和问题。主要是：经济总量不大，经济结构不够合理，工业化、城镇化水平不高；国有企业改革虽然取得明显成效，但难度大进展不快；电力供应不足制约发展问题趋于严重；农业抵御自然灾害的能力不强，结构调整不快，农民增收不多；经济增长点较少，财政收支矛盾突出；城市就业和再就业压力大，部分城乡群众生活困难；重大安全生产事故时有发生等。对上述困难和问题，我们将高度重视，采取有效措施，切实加以解决。

二、2004年工作的总体要求和主要措施

2004年是实现“十五”计划和城市面貌“三年大变”的关键之年，也是加快全面建设小康社会步伐，深化改革、扩大开放、提速发展的重要一年。我们一定要以邓小平理论和“三个代表”重要思想为指导，全面贯彻党的十六大和十六届三中全会，以及市委十届十次全会的精神，坚持以人为本，树立全面、协调、可持续的发展观，深入实施开放带动和项目带动战略，以招商引资为突破口，努力扩大投入，以做大做强工业为主攻方向，调整和优化经济结构，以增加农民收入为重点，加快农业产业化进程，以全力扶持非公经济发展为增长点，培育新的经济主力军，全面推进工业化、城镇化，带旺商贸和旅游业，切实做好就业和再就业工作，不断提高人民群众生活水平，实现我市国民经济持续快速健康发展和社会全面进步。据此，2004年我市经济和社会发展的主要预期目标初步安排为：

——全市生产总值增长9%。

——财政收入增长10%。

——全社会固定资产投资增长20%。

——外贸出口增长10%。

——实际利用外资增长20%。

——引进内资实际到位增长50%。

——规模以上工业企业增加值增长15%。

——全社会消费品零售总额增长10%。

——城镇居民人均可支配收入增长8%，农民人均纯收入增长6%。

——城镇新增就业16000人，城镇登记失业率控制在4.7%以内。

——人口自然增长率控制在7.8‰以内。

虽然完成上述指标，我市的经济也保持一定增长，但必须看到，这样的增速，离我市“十五”期末赶上全区中上水平的要求，还有很大的差距。我们如果不抓住机遇加快发展，就有可能拉大与区内先进市的差距，我们的奋斗目标就有可能落空。面对全国各地、全区各市加速发展的形势和激烈的竞争，我们如同逆水行舟，不进则退，慢进也是退。因此，我们追求完成的任务不仅是上述的预期目标，而是一个更高的奋斗目标。

今年，要重点抓好以下十项工作：

（一）巩固和加强农村经济工作，千方百计增加农民收入

认真贯彻中央和自治区农村工作会议精神，把解决好“三农”问题作为今年各项工作的重中之重。加强农业基础地位，高度重视并认真抓好农业、农村和农民问题，努力发展县域经济。一要严格保护耕地，稳定粮食生产面积和产量。今年全市粮食种植面积要达235万亩，总产量要达88万吨。实行严格的耕地保护制度，坚决遏制乱批乱占和滥用耕地的现象。稳定农村联产承包责任制三十年不变的政策，建立和完善农户依法、自愿、有偿流转土地承包经营权办法。二要巩固和推进农业和农村经济结构调整，发展优质高效农业。在保证粮食总量基本稳定的前提下，支持和引导农民按市场需求调整种养业结构。继续抓好“六大产业”开发和“六带一廊”建设，把畜牧水产业作为农业支柱产业来大力培植和发展。同时做好动物疫病防治和食品安全工作。继续抓好现代农业实验区建设，认真研究和调整园区的经营管理办法，重视高新农业引进和示范推广，努力办出特色，办出成效。三要加大农业投入，加强农村基础设施建设。要抓住今年国债和财政资金向“三农”倾斜的机遇，努力争取国家支持，多方筹措资金，抓好农村水、电、路建设，改善农村的生产生活条件。四要进一步深化农村改革，落实党在农村的各项政策。深化粮食流通体制改革，继续抓好农村税费改革试点工作。推行村务公开，整顿农村市场经济秩序，努力探索加强农村社会保障工作，营造有利于农业和农村经济发展的良好环境。

结合我市农村的实际情况，今年要突出抓好五大措施，促进农村经济的快速发展。一是企业联动措施，今年要在全市范围内选择15个农产品加工企业，作为市、县（市）、区的重点龙头企业，并将之与基地建设、个体农户联合起来，带动农业产业化发展。二是专业村带动措施，在全市选择73个自然村或行政村，按照一村一品的要求发展特色农业，并制定产业发展的目标、规模和任务，定科技部门对口指导，定县乡领导岗位责任，定扶持办法。三是销售队伍拉动措施。全市要扶持、培育61个农产品流通组织和销售大户，对农产品实行定点、定目标、定产品收购，使之与农村经济组织和农民建立牢固的订单、互惠的关系，推动订单农业的发展，为专业村生产出来的农产品提供销售保障。四是劳务驱动措施，建立市、县（区）两级劳务输出领导机构，由县乡（镇）劳动、农业、教育等部门就近免费对农民工进行培训，由市县劳动、经贸、外经、人事、建规等部门推荐就业。通过开展培训，使全市有10万农民受到各种劳动技能培训，有10万农民找到务工机会。五是基础设施推动措施，今年全市共实施基础设施建设项目86项，总投资9716万元，重点解决农村道路、通电和人畜饮水问题，为我市农村经济更快发展创造条件。通过采取上述五项措施，努力使我市今年的农村工作像三百项目大会战一样红红火火，实现农民增收、农村发展的目标。

（二）深化国有企业改革，做大做强重点产业，增加工业总量

梧州提速发展的难点在农业，重点在工业，出路在招商。今年要进一步强化工业的主导地位，集中更大的人力、物力、财力抓工业。根据我市资源和当前财政及企业的状况，要增加工业总量，主要靠外来资金的投入。因此，今年我市工业工作的重点是改制和招商，而且要把招商摆在首位。

突出抓好电力工业建设。发挥我市丰富的水电资源优势和区位优势，大力发展大型水电、火电等电力工业。加快长洲水利枢纽和旺村水电站等水电项目建设，积极争取桂东火电厂落户我市。整合水电资源，加快电网建设，将电力工业迅速提升为我市的支柱产业，使我市逐步成为广西的电力工业基地，成为国家和自治区西电东送的重要支撑。

加大工业投入，做大做强我市食品、日化、药品、电子、宝石、木材加工等主导产业。大力扶持中恒集团、红日公司等企业扩大生产，做大规模。今年计划安排工业项目50项，总投资83.66亿元。其中重点技改项目18项，主要抓好奥奇丽公司和新华公司扩大生产规模易地改造、制药公司GMP改造、蛋白肠衣厂西式低温胶原蛋白肠衣技改、冰泉公司大豆深加工技改项目以及蒙山县变性淀粉项目、万秀区旺甫医药城项目、蝶山区纺织印染项目、长洲区桂新钢铁厂三期技改项目等。抓好蛋白肠衣厂和冰泉公司培育上市工作，力争早日上市。全年工业技改投资要完成5亿元，比上年增长16%。通过技术改造，加快培育形成年销售收入上5亿元的企业2—3家、上8亿元的企业1—2家、上10亿元的企业1家；力争全市拥有纳税超3000万元的企业2—3家、超4000万元的企业1—2家、超5000万元的企业1家。对进入这些行列的企业、集团，要大张旗鼓给予表彰，对为企业做大做强付出努力的有功人员，按梧政发〔2001〕126号文给予重奖。

大力推进国有企业改革，力争全面完成改制任务。要坚持走开门改革、引进外资改造国有企业的路子，充分发挥东晖、东旭两家国有资产经营公司的作用，加大国有企业改制力度，实现我市国有经济占国民经济总量的20%以下，使国有资本尽快从一般性竞争领域退出。改进和简化企业改制方案的审批办法和审批程序，加快审批速度。东晖、东旭公司今年要完成企业改制32家，重点抓好梧锅公司、梧起公司、第一建筑公司、蓄电池厂、光学仪器厂、锻压机床厂、常运电机公司等企业的改制，帮助船用锻铸件厂、合成纤维厂、舞龙公司等企业剥离债务2.5亿元。大力引进资金改造国有企业，争取利用外资700万美元，引进内资3300万元。各县（市）、区也要加快国有企业改革步伐，确保全市今年基本完成国有企业改革任务。

加快发展园区工业。建立健全园区管理体制，全面提高工业园区招商引资竞争力，为工业发展开辟新的发展空间。依法加快土地征用工作，采取多种形式，吸引更多的工业企业进园落户，形成传统优势产业和新引进的优势产业竞相发展的良好局面。今年工业园区存量土地面积要达到2平方公里，落户开工企业达到5家以上，固定资产投资达到1亿元，进园企业实现销售收入3亿元以上。

（三）以招商引资为重点，全方位扩大对外开放

今年是环境建设年、项目年、招商年，我们一定要抓住机遇，全面扩大对外开放，促进经济提速发展。

要发展梧州经济，必须主动将自己融入珠江三角洲经济圈，重新研究审视我市的政策和产业的发展方向，更好地探索与粤港澳合作的最佳方式。不论政策的制定，产业发展的方向，还是企业的嫁接以及招商引资，都要与粤港澳接轨，以谋求更大的发展，壮大我们的实力。要抓住中国——东盟博览会在南宁召开的良好机遇，加强和东盟各国的交流与合作，积极引进东盟企业参与我市项目和产品的开发建设，提高我市对外开放水平。举办梧州首届宝石节，扩大梧州宝石的知名度。

进一步改善投资环境，实行严格的影响投资环境追究制。落实《关于进一步吸引外来投资促进经济发展的岗位责任制》（梧发〔2003〕9号）精神，凡是违反该岗位责任制的要坚决处理。组织有关部门开展联合审批，实行“一个窗口”收费。建立健全外商因服务质量外迁查办制、主动为外商服务检查汇报制及督查制等，强化责任追究，不断提高服务质量。每季度召开一次外商座谈会，检查有关部门的服务情况及外商的满意程度，并加大督查督办力度，进一步转变机关作风，提高办事效率，全面改善投资软环境。

发挥招商专业队伍在招商引资中的主力军作用，采取强有力的招商措施，确保利用外资和引进内资增长目标的实现。招商专业队伍要利用各种形式与大公司、大财团联系，了解他们的发展动向，与他们交朋友，以情动人，以诚感人，千方百计吸引他们到梧州投资。要把招商的主攻方向放在引进新企业，特别是外商独资企业上。市、县（区）领导特别是党委、政府领导要亲自抓招商，发挥模范带头作用，每人要引进1—2个投资规模在1000万元以上的企业。建立招商引资责任制，只要在国家法律和政策允许的范围内，在自治区和我市、县优惠政策的框架内，责任人可自主作出决定，并按规定办理手续、承担责任。

从实际出发，大胆拓宽我市投资领域。在招商引资工作中，要本着“法无禁止即自由”的原则，只要有利于增加我市工业总量，促进我市经济快速发展，就要大力引进各种项目。既要积极争取引进高新技术产业项目和大项目，也要大力引进中小企业，以迅速增加我市工业总量，创造大量的就业岗位。

努力扩大出口。根据国家出口退税政策的变化，及时调整措施，大力培育一批新的外资企业出口加工基地，扶持民营企业发展出口业务，重点支持机电、纺织、宝石、食品等产品的出口。提高外贸出口的服务质量，采取有效措施动员企业自营出口或委托我市外贸企业代理出口业务，确保我市外贸出口目标任务的完成。

（四）继续全力推进三百项目大会战，促进经济提速发展

项目建设是推动我市经济提速发展的关键，是壮大我市经济总量的根本。开展三百项目大会战，是经济提速发展的需要，是提高群众生活水平的需要，是做好就业工作的需要，是赶上全区中上水平的需要。开展三百项目大会战，是我市审时度势作出的重要决策，是今年乃至今后几年我市的一项非常重要的工作。我们要坚定不移，一抓到底。

对三百项目实行动态管理。项目安排总的原则是继续重点抓好我市的产业项目、城市基础设施及交通项目，同时根据统筹兼顾经济与社会、城市与乡村协调发展的原则，重视社会公共服务设施建设，还要上一批农村发展的项目。今年大会战的主要安排是：建设项目100项，总投资约209亿元，当年计划投资约43亿元，其中续建项目58个，新开工项目42个；前期工作项目100项，其中2003年未完成需继续开展的前期工作项目29项，新开展前期工作项目71项，项目总投资158亿元；招商项目100项，投资总额77亿元，预计利用外资49亿元。要突出抓好长洲水利枢纽、南梧高速公路岑溪至梧州段和苍梧至郁南段、西江三桥、苍梧县枫木陂电站、藤县金鸡水利枢纽、南渡至水汶二级公路等重点项目建设，壮大我市经济实力并为发展增强后劲。要扎实推进桂梧高速公路梧州至马江段、洛湛铁路梧州段、旺村电站、梧州新港、西江四桥、苍梧年产120万吨水泥厂等事关全局的重大项目前期工作，符合开工条件的项目，争取尽快开工建设。要继续抓好市区和各县城防洪体系的建设，加强对防洪堤的管理，确保堤防的安全。总之，要通过抓项目来促进经济和社会的提速发展。

要千方百计保证三百项目资金按时足额到位。一方面，要采取盘活资产存量，依法追收财政周转金，调整项目投资结构，向商业银行申请短期贷款等办法，多渠道筹集资金，千方百计解决资金缺口，支持项目建设。另一方面，对工程要实行公开招标，以公开促公正，预防和惩治腐败，抓项目评审，节约开支，尽量多开工一些项目，并根据资金到位情况，适时确定项目开工时间。在资金的使用上要保证重点项目和已开工项目，使重点项目和已开工项目能按时开工、竣工。

继续实行三百项目目标责任制，落实奖罚措施，确保各项目标任务按质按量按时完成。在对完成目标任务者给予奖励的同时，要追究不完成目标任务者的责任。要继续完善和执行项目业主负责制、保证项目落实责任制、领导督办制、首席谈判代表制、招商引资目标责任制等行之有效的制度，及时兑现奖惩，确保三百项目各项任务的完成。

在三百项目建设中，要切实维护群众的利益，妥善处理建设中出现的各种问题，真正把好事办好。要依法征地、拆迁，对征地拆迁户要做好过细的思想工作，要严格按照法律、政策、程序办事，对群众的补偿一定要按照政策给足、给合理。要为群众的生活、生产和发展着想，妥善安置被征地拆迁的群众；要解决好拖欠农民工工资问题，严厉查处拖欠、克扣农民工工资等行为，切实保护农民工的合法权益，今后不允许出现新的拖欠。

（五）加快“三年大变”建设步伐，确保今年目标的实现

“三年大变”主要是指打造一道具有梧州特色的沿江风景线，再构造文澜、富民、西堤等精品路，建设文化广场、图书馆、博物馆、体育中心等标志性建筑，改造骑楼城、地质灾害居民区、主干道建筑立面以及白云山公园、中山公园、中山纪念堂等景观，高标准建设红岭片路网。

今年市政建设的总体要求和目标是，改造河西片路网，续建并完成新兴三路延长线、骑楼城改造、鸳江丽港、西堤路拓宽、地质灾害整治、河东主要街道立面装饰、拆迁安置小区等工程。新开工建设新兴路与西堤路连接道路改造工程、红岭片路网工程、文化广场、中山路和文化路改造、升龙秀湾等项目，力争河东、河西片环境综合整治工程今年开工并竣工。同时，要加强城市管理，严格执行城市管理法规，以市容综合治理和交通秩序整治为突破口，下大力气改变城市“脏、乱、差”的现象，努力为市民创造文明、整洁、美丽的环境。

要实现上述目标，必须坚持规划先行，充分发挥规划的龙头作用。要求能开工的尽可能提早开工；要加强施工现场管理，保证质量；要多引进外地有实力的施工队，参与我们的各项建设。要有效调动各方力量，吸纳各类资金，参与城市建设。同时，要树立经营城市的理念，提高城市资源利用效益。继续开展建设用地清查，依法收回征而未用的土地。采取切实有效的措施加快征地进度，做好土地储备工作，今年的土地储备要达5000亩。

（六）加强财税工作，确保财政金融平稳运行

要把支持经济发展，支持开放带动、项目带动两大战略的实施作为财政工作的重要任务，努力培植财源。市、县（市）、区财政今年安排3992万元用于企业改革和技术改造，支持企业技术创新和新产品研发，支持工业化和城镇化建设，安排3850万元用于农业投入，支持农村基础设施建设，支持农业和农村经济结构调整，发展农村经济，促进农民增收，在此基础上，还要从财政挤出部分资金支持三百项目建设，培植一批新的经济增长点。市政建设除安排开发行的15亿贷款外，京南电站、鸳江大桥和公房转让以及土地出让的纯收入等也主要用于市政建设。

进一步加强税收征管，确保财政收入稳定增长。坚决制止擅自批准减税、免税、缓税和先征后返还的违规行为，严格执行优惠政策的审批制度和权限。依法加大对税收的监缴和稽查力度，努力做到依法征税、应收尽收，坚决完成财政收入目标。要确保法定支出和政策性重点支出，首要的是保证工资按时足额发放和社会保障需要，不能出现新的拖欠，各县（市）、区要千方百计发放适当生活补贴。支持教育、科技、文化、公共卫生、体育等社会事业发展。积极推进财政各项改革，切实加强对非税收入的管理，严格执行“收支两条线”。逐步建立和完善公共财政体制的基本框架，优化财政支出结构，强化支出效益。继续深化部门预算改革，加快推进国库集中收付。完善政府采购制度。进一步强化财政管理和监管，整顿和规范财经秩序。

加强对三百项目资金监管的力度，建立和完善项目责任制，组织专门人员对每个项目进行评估，确定有收益项目的偿还额度、时间，办理偿还抵押的相关手续，并确定偿还责任人和逾期不还的责任追究制，确保资金回收。支持金融改革，加强金融监管和金融服务。规范社会信用秩序，努力培育良好的信用环境。不断拓宽融资渠道，积极争取银行增加梧州经济和社会发展的有效信贷投入。继续抓好“两会一部一借”和城市信用社清理整顿工作，消除经济金融隐患，防止和化解金融风险。

（七）进一步壮大非公经济，努力打造我市经济的主力军

要把发展非公有制经济摆在更加突出的位置，采取更有力的措施，促进非公有制经济更快更好地发展，使其早日成为我市经济发展的主力军，大幅度提高非公有制经济在我市国民经济中的比重。一是各级政府要把推动非公有制经济大步发展作为一项重要任务来抓。要牢固树立抓非公有制经济就是抓发展的思想，按照“政治平等、政策公平、法律保障、放手发展”的方针，落实各项政策，依法提供保护，大力优化环境，努力改进服务，尽快把外商投诉、外来投资服务、外来投资进出口服务等“三个中心”改为为非公经济服务的“三个中心”，认真解决非公有制经济发展中遇到的实际问题，积极推动和引导非公有制经济健康发展。二是下大决心扫除制约非公有制经济发展的体制障碍。要放宽市场准入，凡是已向外资开放或承诺开放的投资领域，都向非公有资本开放，凡是国家法律法规没有禁止进入的行业和领域，都允许非公有资本进入。要消除政策歧视，使非公有制企业与其他企业一样享受各种同等待遇。三是要制定和完善扶持非公有制经济发展的相关政策，加大对非公经济的财政、税收、用地、融资、劳动保障等方面的支持，扶持非公经济做大做强。四是要在今年内实现非公有制经济占我市国民经济总量的比重达80%以上，必须完成国有企业的改制任务，必须引进大量的非公有制经济企业，必须支持现有非公有制经济企业发展壮大，必须停止新办国有独资企业。

加快发展旅游和服务业。充分发挥梧州的地缘优势，利用粤港澳——梧州——桂林这条黄金旅游线，加快开发我市的旅游景区、景点，打造旅游精品。加快白云山风景区、中山公园、龙母太庙、太平天国封王遗址、太平狮山、黄华河吉太景区等重要景点的建设和改造。积极开展旅游促销活动，通过发展旅游，增加人气，带动三产，带旺城市。重视和抓好服务业的发展，促进商贸大流通。充分发挥区位和交通组合优势，加快物流设施平台、信息平台和政策平台建设，构建独具特色、有竞争力的综合物流体系。发展新型商业，建设各类专业市场。

（八）做好就业和社会保障工作，改善人民生活

今年要把就业和再就业工作放在更加突出的位置抓紧抓好，各级党政一把手要高度重视就业和再就业工作，努力解决影响就业和再就业工作的重点难点问题。一是要进一步强化工作目标责任制，将增加就业人数、帮助下岗失业人员特别是大龄失业人员实现再就业和控制城镇登记失业率等各项指标层层分解、层层落实，作为政绩考核的重要内容。二是要进一步加大就业和再就业政策的落实力度。各县（市）、区，各部门要不折不扣地将税费减免、税收优惠、社保补贴、小额贷款、免费培训等各项再就业扶持政策落到实处，不能以任何借口打折扣或不执行。三是要进一步抓好再就业资金的到位。建立和完善促进再就业专项基金，积极运用财政、税收、信贷等手段加大投入，并充分调动社会各方面投入的积极性，努力确保资金的落实。四是要进一步加大就业岗位开发力度，采取各种措施拓宽就业渠道，千方百计扩大就业。要抓好公益性岗位开发工作，今年市、县（市）、区政府都要拿出一定的资金购买1200个以上公益性岗位安排下岗职工，尤其是“4050”人员就业。继续办好下岗失业人员再就业交流大会。五是要进一步加大就业培训力度，提高劳动者素质。今年市、县（市）劳动部门要免费培训下岗职工和失业人员1.2万人次，使受训人员达到初级或中级技能水平，提高其再就业的竞争力。

建立健全与经济发展水平相适应的社会保障体系。扩大社会保险的覆盖面，为人民群众构筑生活“安全网”。认真落实“两个确保”和城市“低保”工作，重视抓好国有企业下岗失业人员基本生活费、离退休人员养老金的按时足额发放。抓好城镇居民最低生活保障，做到应保尽保。基本完成全市城镇企业退休人员纳入社区管理服务，进一步完善医疗保险制度，切实解决困难企业参加医保问题。

坚持以人为本，切实关心群众生活。要特别关注弱势群体生活困难问题，采取有效措施，切实保障社会特殊群体和特困家庭的基本生活。着力帮助老复员军人、下岗职工、农村特困户、五保户、劳动模范等解决实际困难，切实保障他们的基本生活。

（九）坚持实施科教兴市和可持续发展战略，促进社会各项事业发展

高度重视科技事业发展。加强信息化建设，努力以信息化带动和促进工业化。大力推动科技创新，积极开展以产品创新为核心的技术创新工作，全力构建重大科技项目，精心培育高新产品，铸造科技品牌，建立技术市场，推进科技与经济相结合。增加教育投入，整合教育资源，努力提高教育质量。全力抓好西大分校申报本科院校工作，解决好软硬条件问题，志在必得。加快市十五中的建设步伐，坚持高标准建校，力争今年秋季招生。积极落实好资金，要在完成去年10万平方米中小学危房改造任务的基础上，今年再增加8万平方米的改造任务，实施办法仍然是争取上级专款、财政拨款和部门支持相结合。鼓励社会力量办学，发展职业教育，逐步完善多元化办学体系。加强各级各类人才的培养，优化人才成长环境，落实各项政策措施，充分利用好现有人才，吸引高层次急需人才。

促进卫生、文化、体育等其他社会各项事业发展。加强公共卫生基础设施建设，建立健全公共卫生体系，提高公共卫生服务水平和突发性公共卫生事件应急能力。推进城镇医疗卫生体制改革。继续抓好市区医院资源整合，提高利用效率。着力发展农村医疗卫生事业，逐步建立和完善新型的农村合作医疗制度。落实措施抓好非典型肺炎防治工作，市传染病医院要按时在今年3月投入使用。继续加大基层文化基础设施建设，积极培育和发展文化产业。加强文化对外交流，精心准备我市首次出国演出的节目，确保演出成功。广泛开展全民健身运动，提高竞技体育水平，办好今年梧州市第九届运动会。积极发展体育产业，重点培育和发展体训基地，抓好体育中心的建设。进一步密切军政军民关系，巩固双拥工作成果。加强人口、资源和环境保护工作，重视和抓好计划外生育反弹和出生人口性别比偏高及流动人口计划生育管理难问题，以改善环境质量和保障人民健康为根本，实施可持续发展战略，实现经济和社会、人和自然的协调发展。

（十）进一步转变政府职能，提高经济社会管理水平

转变政府职能，建立服务型政府，努力提高服务质量。要继续推进政企分开、政资分开、政事分开，把政府不该管的事交给企业、市场、社会组织和中介机构，更大程度地发挥市场在资源配置中的基础性作用，增强整个社会经济的活力与效率。加快推进政府审批制度改革，使行政审批走向制度化、规范化和法制化，实现政务公开。要更加重视履行社会管理和公共服务职能，把财力、物力等资源更多地用于社会管理和公共服务，把精力更多地放在推进社会发展和解决群众生活问题上，着力建设服务型政府。

切实提高依法行政水平。今年7月1日正式生效的《中华人民共和国行政许可法》是促进政府职能转变，依法行政，规范政府行政行为的重要法律。我们要认真组织好《行政许可法》的学习、宣传和培训，保证《行政许可法》顺利实施。要做好对文件、实施行政许可主体的清理工作，凡是与《行政许可法》相违背的，一律废止或修改。所有政府工作人员都要依法处理经济社会事务，提高行政管理水平。严格实行行政执法责任制和执法过错追究制，真正做到“有权必有责、用权受监督、侵权要赔偿”，全面营造依法行政的良好政务环境。加强民主法制建设。各级政府要自觉接受同级人民代表大会及其常委会的监督，支持人民政协工作，广泛听取社会各方面的意见，认真办理人大代表议案、建议、批评、意见和政协委员提案。

全力维护社会稳定，优化发展环境。继续深入开展市场专项整治，严厉打击扰乱市场经济秩序的各种经济违法违章行为。建立企业信用制度，促进企业守法经营。建立规范的个人信用联合征信制度，提高公民诚信意识。加强社会治安防范，深入开展“严打”斗争，严厉打击各种刑事犯罪和经济犯罪活动，严密防范和严厉打击境内外敌对势力、“法轮功”等邪教组织及暴力恐怖势力的渗透破坏活动，坚决打击城乡各种赌博尤其是“六合彩”赌博活动，保障人民安居乐业。解决好社会热点、难点问题，妥善处理人民内部矛盾。重视群众来信来访工作，切实把矛盾和问题解决在基层，化解在萌芽状态，维护社会稳定。切实加强安全生产监督管理，进一步落实安全生产责任制和特、重大安全事故行政责任追究制度。抓好安全生产专项整治，排除事故隐患，遏制安全生产事故发生，今年一定要把重大事故发生率降下来。

坚持求真务实的工作作风，多为群众办实事。各级政府要强化执政为民意识，把群众的呼声作为第一信号，把群众的需要作为第一选择，把群众的利益作为第一考虑，把群众的满意作为第一标准，实现好、维护好、发展好人民群众的根本利益。要注重了解民情、反映民意、集中民智、珍惜民力，把心思和精力放在诚心诚意、勤勤恳恳为人民服务上。办实事要讲实效，集中精力多为群众搞一些民心工程、德政工程，做到权为民所用，情为民所系，利为民所谋。为民办实事好事，要从实际出发，量力而行，能办的大事一定要办，因财力问题一下办不了大事的，小事也不能忽视。绝对不能大事办不了，小事又不办。深入开展廉政建设和反腐败斗争，加强对公务人员的教育、管理和监督，发扬艰苦奋斗作风，增强拒腐防变能力。加强机关效能建设，不断提高办事质量和效率，努力使各级政府成为人民群众满意的政府。

各位代表！我们正处在加快发展的重要时期，任务艰巨、使命重大。让我们紧密地团结在以胡锦涛为总书记的党中央周围，高举邓小平理论伟大旗帜，全面贯彻“三个代表”重要思想，认真落实党的十六大和十六届三中全会，以及市委十届十次全会精神，进一步解放思想，奋发图强，开拓进取，扎实工作，全面完成我市今年经济和社会发展各项任务，为实现梧州经济提速发展和全面建设小康社会而努力奋斗！
